# *In Vivo* Activity of Amodiaquine against Ebola Virus Infection

**DOI:** 10.1038/s41598-019-56481-0

**Published:** 2019-12-27

**Authors:** Lisa Evans DeWald, Joshua C. Johnson, Dawn M. Gerhardt, Lisa M. Torzewski, Elena Postnikova, Anna N. Honko, Krisztina Janosko, Louis Huzella, William E. Dowling, Ann E. Eakin, Blaire L. Osborn, Janet Gahagen, Liang Tang, Carol E. Green, Jon C. Mirsalis, Michael R. Holbrook, Peter B. Jahrling, Julie Dyall, Lisa E. Hensley

**Affiliations:** 1 Integrated Research Facility, National Institute of Allergy and Infectious Diseases, National Institutes of Health, Frederick, MD 21702 USA; 2 Present Address: Emergent BioSolutions Inc, Gaithersburg, MD 20879 USA; 3 Present Address: AbViro LLC, Bethesda, MD 20814 USA; 4 Present Address: Bioqual Inc, Rockville, MD 20850 USA; 5 Present Address: Vaccine Research Center, National Institute of Allergy and Infectious Diseases, National Institutes of Health, Bethesda, MD 20892 USA; 6 Division of Microbiology and Infectious Diseases, National Institute of Allergy and Infectious Diseases, National Institutes of Health, Rockville, MD 20892 USA; 7 SRI International, Menlo Park, CA 94025 USA; 8 Emerging Viral Pathogens Section, National Institute of Allergy and Infectious Diseases, National Institutes of Health, Frederick, MD 21702 USA

**Keywords:** Ebola virus, Viral infection

## Abstract

During the Ebola virus disease (EVD) epidemic in Western Africa (2013‒2016), antimalarial treatment was administered to EVD patients due to the high coexisting malaria burden in accordance with World Health Organization guidelines. In an Ebola treatment center in Liberia, EVD patients receiving the combination antimalarial artesunate-amodiaquine had a lower risk of death compared to those treated with artemether-lumefantrine. As artemether and artesunate are derivatives of artemisinin, the beneficial anti-Ebola virus (EBOV) effect observed could possibly be attributed to the change from lumefantrine to amodiaquine. Amodiaquine is a widely used antimalarial in the countries that experience outbreaks of EVD and, therefore, holds promise as an approved drug that could be repurposed for treating EBOV infections. We investigated the potential anti-EBOV effect of amodiaquine in a well-characterized nonhuman primate model of EVD. Using a similar 3-day antimalarial dosing strategy as for human patients, plasma concentrations of amodiaquine in healthy animals were similar to those found in humans. However, the treatment regimen did not result in a survival benefit or decrease of disease signs in EBOV-infected animals. While amodiaquine on its own failed to demonstrate efficacy, we cannot exclude potential therapeutic value of amodiaquine when used in combination with artesunate or another antiviral.

## Introduction

Currently, no vaccines, therapeutics, or prophylactics are approved to prevent or treat disease resulting from Ebola virus (EBOV) infection. The two largest recorded outbreaks of Ebola virus disease (EVD), which include the recent 2013–2016 Ebola virus outbreak in Western Africa and the ongoing outbreak in the Democratic Republic of Congo, illustrate the critical need for the development and approval of medical countermeasures. These countermeasures could potentially treat patients with suspected EVD, prevent the development of disease following a known exposure, or protect those at risk for possible exposure, such family members or health care workers and laboratory support staff working to control EVD outbreaks.

Patients with suspected EVD are provided supportive care and standard therapies to treat other infections with similar onset of symptoms (e.g., malaria, typhoid fever) prevalent in the area^1^. The World Health Organization recommends that patients with suspected EVD are treated with an antimalarial empirically when malaria diagnostics are not available or when test results are delayed^1^. In August of 2014, an Ebola treatment center in Liberia was in short supply of the standard antimalarial combination drug (artemether-lumefantrine) used for patients with suspected EVD. As a result, 71 patients with confirmed EVD were prescribed the antimalarial combination drug which contains the compounds artesunate (AS) and amodiaquine (AQ). A recent study evaluated and compared the outcome of these EVD patients who were prescribed artesunate-amodiaquine (ASAQ) with those who received artemether-lumefantrine^2^. Patients who were prescribed ASAQ had a 31% lower risk of death relative to a group of 191 patients treated with artemether-lumefantrine. All patients were admitted into the same treatment center within a 5-month span, had confirmed EVD, and had no other obvious changes in their care during this period. Another retrospective study performed on data from 5 Ebola treatment centers in Liberia and Sierra Leone also showed a trend towards decreased mortality in EVD patients that received ASAQ^3^. As both artemether and AS are derivatives of artemisinin, the potential beneficial anti-EBOV effect observed with ASAQ could possibly be attributed to the change from lumefantrine to AQ. Alternatively, the improved response might be attributed to adverse effects of lumefantrine in EVD patients rather than improved treatment.

AQ has been identified as a potent EBOV inhibitor in multiple *in vitro* assays^4–8^. AQ is rapidly metabolized to the active metabolite, desethylamodiaquine (DEAQ), following oral administration. Both AQ and the metabolite DEAQ are known to have *in vitro* antiviral activity against EBOV^8^. AS has been reported to have antiviral activity against other viruses^9^, but no activity against EBOV has been demonstrated^4^. While AQ *in vitro* activity against EBOV has been characterized in detail, evaluation in a more relevant disease model, such as nonhuman primates (NHPs), has not been performed.

AQ was not efficacious against mouse-adapted EBOV (ma-EBOV) in mice^7^; however, the dose (60 mg/kg BID) and route of administration (intraperitoneal) were different from the treatment regimen used in human patients. In addition, the EBOV infection mouse model using ma-EBOV does not mimic aspects of human disease, such as rash, coagulation, and hemorrhagic manifestations. Therefore, countermeasures for the treatment or prevention of EBOV infection should be appropriately evaluated in relevant animal models prior to being used in humans. Both the cynomolgus and rhesus macaque models have previously been shown to mimic many critical aspects of human filovirus disease^10^.

Here, we evaluated the efficacy of AQ in the rhesus macaque model of EVD when orally administered using a similar dosing regimen to that used in human patients. AQ was administered as a consecutive 3-day course of 20 mg/kg free base (26 mg/kg amodiaquine hydrochloride). Treatment began on the day of EBOV exposure or on day 3 postexposure. This study found that AQ had no *in vivo* efficacy for the treatment of EVD in rhesus macaques.

## Results

### Antiviral activity of amodiaquine and artesunate and their metabolites in cell culture

The antimalarial treatment ASAQ that was administered to EVD patients in 2014, is a coformulation of artesunate (AS) and amodiaquine (AQ). AS and AQ are rapidly metabolized in the liver to dihydroartemisinin (DHA) and desethylamodiaquine (DEAQ), respectively. Prior to *in vivo* evaluation, the drug ASAQ, its components, and the metabolites were characterized for their inhibitory effects on EBOV (Makona variant, EBOV/Mak) replication in cell culture (Table 1). The data confirm previous reports that both AQ and the metabolite DEAQ block EBOV replication with similar activity (IC_50_ = 2.8 to 3.2 µM) in Huh 7 and Vero E6 cell lines (IC_50_ = 9.5 to 11 µM). In primary human macrophages, both AQ and DEAQ exhibited elevated cytotoxicity, which precluded any interpretation of antiviral activity. In contrast, activity of AS and its metabolite DHA was weak or undetectable. Combinatorial testing of AQ and AS or the metabolites, DEAQ and DHA, did not reveal *in vitro* synergistic effects against EBOV replication (data not shown). Based on the *in vitro* data, the decision was made to evaluate AQ in the NHP model of EBOV infection.

**Table 1:** Antiviral activity of amodiaquine, artesunate, and their metabolites in different cell types.

Drug name	Cell type	Ebola virus variant	CC_50_ (µM)	IC_50_ (µM)^a^	SI^b^	Max effect (µM)
Amodiaquine dihydrochloride hydrate	Vero E6	Makona	40.1	9.5 ± 1.0	4.2	100% at 30
	Huh7	Makona	22.7	3.2 ± 0.03	7.1	100% at 40
	MDM	Makona	20.5	27.9 ± 4.3	0.7	40‒80% at 40
Artesunate	Vero E6	Makona	>40	N.D.	N.A.	25.3% at 40
	Huh7	Makona	>40	17.2 ± 1.2	>5.1	64.3% at 40
	MDM	Makona	>40	N.D.	N.A.	46.5% at 40
Artesunate/Amodiaquine (Winthrop)	Vero E6	Makona	32.9	6.3 ± 1.0	5.2	100% at 30
	Huh7	Makona	15.4	3.0 ± 0.03	5.1	100% at 40
	MDM	Makona	17.3	20.8 ± 0.5	0.8	100% at 40
N-Desethylamodiaquine hydrochloride	Vero E6	Makona	33.1	11 ± 0.6	3.0	100% at 40
	Huh7	Makona	17.8	2.8 ± 0.5	6.4	100% at 40
	MDM	Makona	14.1	30 ± 9.9	N.A.	80% at 40
Dihydroartimesinin	Vero E6	Makona	>40	31.5 ± 1.4	>1.3	64% at 40
	Huh7	Makona	>40	35.2 ± 4.7	>1.1	49% at 40
	MDM	Makona	N.D.	N.D.	N.D.	N.D.

^a^IC_50_ values are mean values ± standard deviation from 2 to 4 dose response curves; ^b^SI = CC_50_/IC_50_; concentrations (CC_50_, IC_50_) are based on the amodiaquine base component of the drug. MDM, monocyte-derived macrophages; N.A., not applicable; N.D., not determined—no activity or not enough data points.

### Pharmacokinetics of amodiaquine in nonhuman primates

The objective of the study was to treat NHPs with an AQ dosing regimen similar to that used for EVD patients in Ebola treatment centers in 2014. Treatment consisted of a 3-day course of AS-AQ with the dose determined according to age of the patient. The dose range for AQ in humans is 7.5 to 15 mg/kg corresponding to a rhesus macaque equivalent dose range of 23.3 to 46.5 mg/kg based on body surface area^11^.

**Figure 1:**
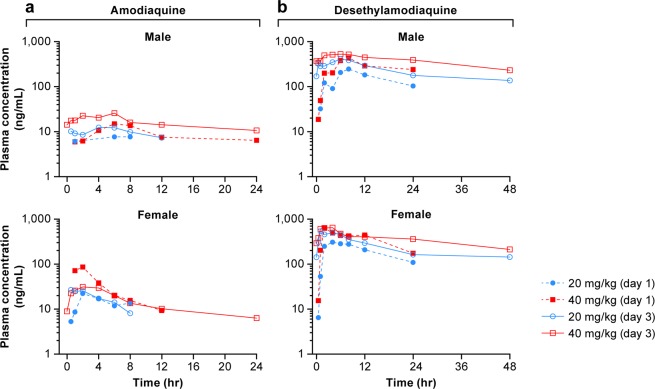
Plasma concentrations of amodiaquine and its metabolite desethylamodiaquine in healthy rhesus macaques. Amodiaquine was administered at 20 or 40 mg/kg for 3 consecutive days. Amodiaquine (**a**) and desethylamodiaquine (**b**) concentrations in the plasma were determined at indicated time points following dosing on day 1 and day 3.

A pharmacokinetic (PK) study in rhesus macaques (2 groups of 2 males and 2 females) was performed to monitor plasma concentrations of AQ (Fig. 1a) and the active metabolite DEAQ (Fig. 1b). The study evaluated three daily oral doses of AQ, using 20 mg/kg or 40 mg/kg. Pharmacokinetics were determined by collecting blood samples at 0, 0.5, 1, 2, 4, 6, 8, 12, 24, and 48 hours (h) following dosing on day 1 and day 3 (Fig. 1). After 3 days of dosing with 20 mg/kg in healthy male and female macaques, AQ was well-tolerated. The higher dose of 40 mg/kg resulted in a variety of clinical observations including hypoactivity, shivering (muscle tremors) and/or diarrhea. Extreme hypoactivity was observed in a single male of the 40 mg/kg group that resolved after dosing ceased. Animals in both the 20 mg/kg and 40 mg/kg groups had lower blood pressure readings than at predose on both dosing days, typically at 4 and/or 6 h post-dosing. Clinical chemistry and hematology parameters for both doses were analyzed and fell within normal ranges with the exception of liver enzymes. Alanine aminotransferase (ALT) and aspartate aminotransferase (AST) were elevated 2.6- to 6.3-fold on day 4 at 24 h after the 3^rd^ dose. The increases in ALT and AST were seen in both male and female animals at both the 20 and 40 mg/kg dose regimens, though changes were not dose-related. These increases were considered toxicologically significant.

AQ and the metabolite DEAQ reached peak concentrations at 0.5 to 8 h (T_max_) after administration for both day 1 and day 3 dosing (Supplementary Tables 1 and 2). The elimination phase half-life (t_1/2_) varied among the individual animals, ranging from 3 to 28 h for AQ and 12 to 51 h for DEAQ. Plasma concentrations of the metabolite DEAQ were higher than concentrations of the parent drug resulting in AUC_last_, and AUC_inf_ values that were 2.5- to 3-fold higher for DEAQ than for AQ. The 20 mg/kg AQ dose resulted in C_max_ for AQ (7.8‒31.9 ng/mL) and DEAQ (203‒369 ng/mL) that were in a similar range as reported for the 10 mg/kg dose in humans (AQ: C_max_ = 29.2 ± 10.9 ng/mL and DEAQ: C_max_ = 268.7 ± 70.8 ng/mL)^12^.

### Amodiaquine did not improve outcome of Ebola virus infection in nonhuman primates

Based on the results of the pharmacokinetics study, the 20 mg/kg AQ dose was chosen for a study to evaluate the *in vivo* effect of AQ in the NHP model of EVD (Study outline, Supplementary Fig. 1). Rhesus macaques were challenged intramuscularly (IM) with a target dose of 1000 plaque-forming units (PFU) of EBOV/Mak (measured dose of 1080 PFU) on study day 0. The control group 1 (n = 3, 1 female, 2 males) received vehicle treatment on days 1, 2, 3, 4, and 5 postexposure. Treatment group 2 (n = 6, 3 females, 3 males) received a 3-day course of 20 mg/kg of AQ by oral administration on days 0, 1 and 2 after exposure to EBOV. Treatment group 3 (n = 6, 3 females, 3 males) received a 3-day course of 20 mg/kg of AQ by oral administration on days 3, 4, and 5 after exposure to EBOV.

**Figure 2:**
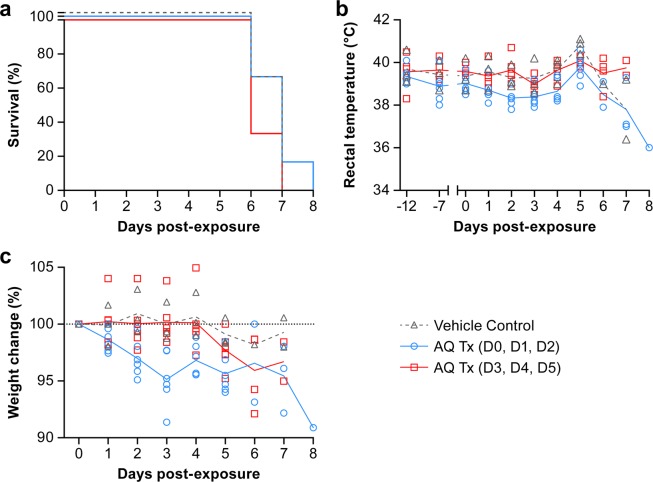
Effect of amodiaquine treatment in animals infected with Ebola virus. Rhesus macaques (3 animals in group 1, 6 animals each in groups 2 and 3) were exposed IM to 1,080 PFU EBOV Makona variant and received either vehicle control (group 1, black) or amodiaquine treatment on days 0, 1, and 2 (group 2, blue) or on days 3, 4 and 5 (group 3, red). (**a**) Percent survival of EBOV-infected treated rhesus macaques (blue and red) compared to vehicle control (black). (**b**) Rectal temperature and changes in weight (**c**) of treated (blue and red) and untreated (black) EBOV-infected NHPs throughout the study.

All animals were euthanized or succumbed to disease by day 8 postexposure (Fig. 2a). Median times to disposition were 7 days for placebo and treatment group 2, and 6 days for treatment group 3 with no significant difference between the placebo or treatment 2 groups versus the treatment group 3 (*p* = 0.2120 and 0.3711, respectively). The animals in all groups became febrile by day 4 or 5 postexposure, followed by a marked decrease in body temperature preceding death (Fig. 2b). No significant difference in febrile illness or weight loss was observed between control and treatment groups (Fig. 2b,c). NHPs had a mild reduction in activity and responsiveness by day 3 postexposure with a severe decrease observed for most animals on day 6 postexposure (Table 2). Loss of appetite and lymphadenopathy began on day 4 to 5 followed by dehydration and rash (petechial, maculopapular) on day 6 postexposure. Clinical signs did not differ significantly between the groups.

**Table 2:** Clinical scores for responsiveness of animals.

Group	Animal No.	Day 0–2	Day 3			Day 4		Day 5			Day 6			Day 7				Day 8			
		AM	AM	PM	Late	AM	PM	AM	PM	Late	AM	Mid	PM	AM	Mid	PM	Late	AM	Mid	PM	Late
1	NHP 01	0	0	0	0	0	0	0	0	0	0	0	1	3	4						
1	NHP 09	0	0	1	0	0	0	0	0	0	3	4									
1	NHP 10	0	0	0	0	0	0	0	0	0	1	1	1	2	2	3					
2	NHP 06	0	1	1	0	0	0	1	1	2	2	2									
2	NHP 08	0	1	2	1	1	1	2	2	2	3	3	3								
2	NHP 11	0	1	2	1	1	1	1	1	1	1	1	2	2	2	2	3	4			
2	NHP 12	0	0	0	0	0	0	0	0	0	0	0	1	3	3						
2	NHP 13	0	1	2	1	0	0	0	1	0	1	1	1	1	1	3					
2	NHP 15	0	1	2	1	1	1	1	2	1	1	1	2								
3	NHP 02	0	0	0	0	0	0	0	0	0	0	0	2	2	2	3					
3	NHP 03	0	0	0	0	0	0	0	0	0	1	1									
3	NHP 04	0	0	0	0	0	0	0	0	1	4										
3	NHP 05	0	0	0	0	0	0	0	0	0	1	1	1	3	3	3	3				
3	NHP 07	0	0	0	0	0	1	1	2	2	2	2	4								
3	NHP 14	0	0	0	0	0	0	0	0	1	3	4									

Responsiveness of unanesthetized animals was scored using the following criteria: 0 (white) = alert, responsive, normal activity, free of disease signs or exhibits only resolved/resolving disease signs; 1 = slightly diminished general activity, subdued but responds normally to external stimuli; 2 = withdrawn, may have head down, fetal or hunched posture, or reduced response to external stimuli; 3 = recumbent but able to rise if stimulated, or moderate to dramatically reduced response to external stimuli; 4 = persistently recumbent, severely or completely unresponsive, or may have signs of respiratory distress.

### Viral loads in plasma were not reduced in treated animals

**Figure 3:**
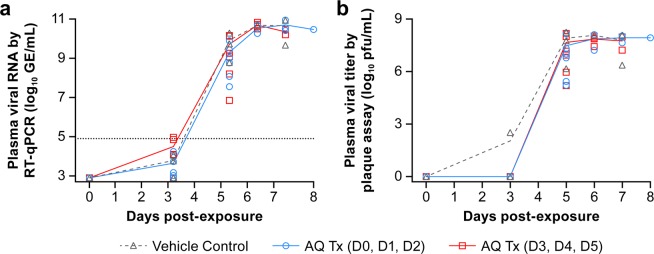
Viral loads in plasma of amodiaquine- and placebo-treated Ebola virus-infected animals. Animals were treated orally with placebo (group 1, black), with 20 mg/kg amodiaquine either on days 0, 1, 2 (group 2, blue) or on days 3, 4, 5 (group 3, red). Viremia was measured by quantitative RT-qPCR (**a**) and plaque assay (**b**).

Plasma viremia was quantified by RT-qPCR, and viral titer was determined by plaque assay. Viral RNA was first detected in the serum of some animals 3 days after exposure, with a rapid and substantial increase by day 5 for both placebo- and AQ-treated animals. RNA levels at necropsy were in the range of 10^9^–10^11^ viral RNA copies/ml for all NHPs (Fig. 3a). Infectious viral titers followed a similar trend for all groups with a sharp increase in viral titer starting on day 3 postexposure and reaching a peak plateau by day 5 before animal death (Fig. 3b). Viremia correlated with the onset of clinical signs of disease such as loss of appetite, lymphadenopathy, and fever. AQ treatment did not reduce plasma viral RNA copies or infectious virus titer regardless of when treatment was initiated. No significant difference in viral titers was observed between groups.

### Amodiaquine-treated animals present with abnormal hematology and biochemical profiles and pathological disease that corresponds with Ebola virus disease

**Figure 4:**
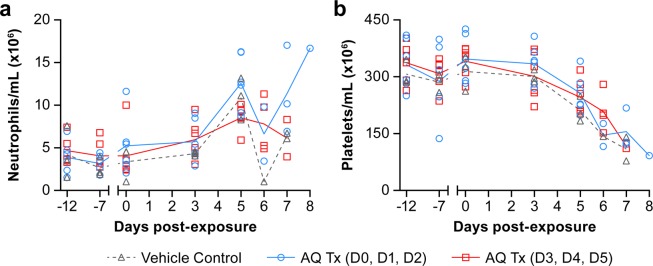
Neutrophil and platelet levels in amodiaquine- and placebo-treated Ebola virus-infected animals. Animals were treated orally with placebo (group 1, black), with 20 mg/kg amodiaquine either on days 0, 1, 2 (group 2, blue) or on days 3, 4, 5 (group 3, red). On the indicated days, neutrophil (**a**) and platelet counts (**b**) were determined. Results are presented as individual data points with the group means connected by a line.

**Figure 5:**
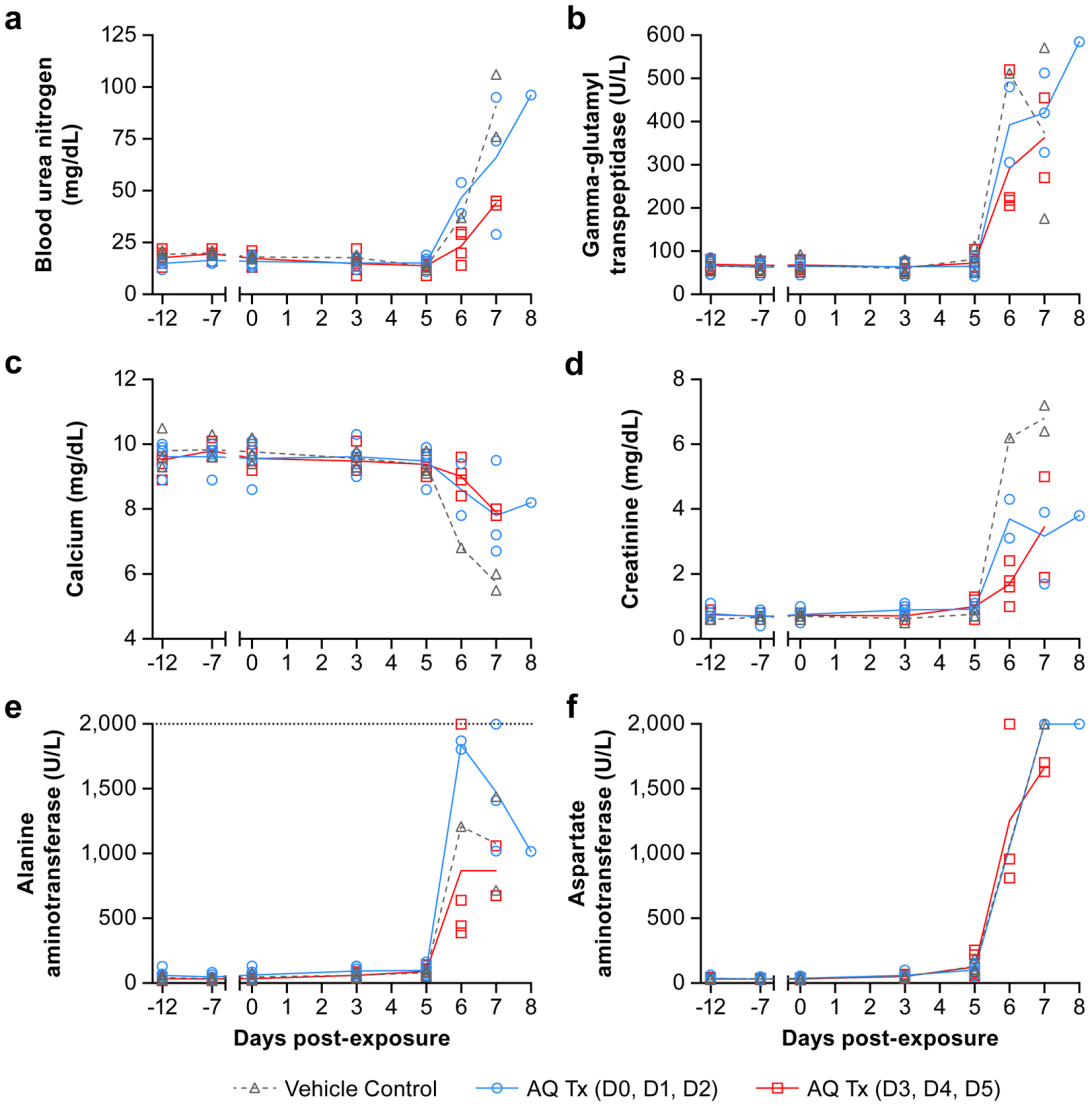
Serum chemistries of EBOV-infected rhesus macaques. Animals were treated orally with placebo (group 1, black), with 20 mg/kg amodiaquine either on days 0, 1, 2 (group 2, blue) or on days 3, 4, 5 (group 3, red). On the indicated days, the following chemistry values were determined in the serum: blood urea nitrogen (**a**), gamma-glutamyl transpeptidase (**b**), calcium (**c**), and creatinine (**d**), alanine aminotransferase (**e**), and aspartate aminotransferase (**f**). Results are presented as individual data points with the group means connected by a line.

The animals showed a similar hematological profile regardless of treatment. Complete blood counts and serum chemistry analysis were performed at scheduled sampling points, before (days -12, -7, and 0) and after (days 3, 5, 7, and at necropsy) exposure (Figs. 4, 5). The chemistry values for AQ-treated NHPs followed the same trends as the control animal values. Similar hematological and biochemical profiles were observed for all animals regardless of treatment both in AQ- and placebo-treated animals. Concurrent neutrophilia and thrombocytopenia were observed for AQ- and placebo-treated animals on day 5 postexposure with EBOV (Fig. 4). As animals reached endpoint criteria, neutrophil levels decreased (Fig. 4a). The low-level neutrophil values on day 6 postexposure were from necropsy samples only. NHPs from all treatment groups exhibited a concomitant increase in serum creatinine and blood urea nitrogen levels, indicative of renal dysfunction often noted in NHPs with concurrent EVD (Fig. 5a,d). Other biochemical abnormalities consistent with EVD were also observed beginning on day 6 postexposure, including substantial elevation of serum ALT, AST, and gamma-glutamyl transpeptidase (GGT) levels that indicate hepatocellular damage (Fig. 5b,e,f).

Necropsies were performed on all animals to evaluate pathological disease associated with EBOV infection following AQ or placebo treatment. The pathologist was blinded to the group identification during necropsy and tissue evaluation. The pathological findings were similar in type and severity between treated and untreated animals, and the gross and histopathologic findings in all animals were consistent with typical EVD in rhesus macaques. Common gross findings included a cutaneous rash on the face, often extending to other regions of the body, dehydration, discoloration of the liver with increased friability, turgid spleen, and enlarged, often congested kidneys. At the microscopic level, all animals exhibited lymphoid depletion of the axillary lymph nodes, lymphoid depletion and necrosis of the spleen, and hepatocellular degeneration and necrosis. Panniculitis and necrosis were observed at the virus challenge site of 6 animals.

### Pharmacokinetics of amodiaquine in Ebola virus-infected animals

**Figure 6:**
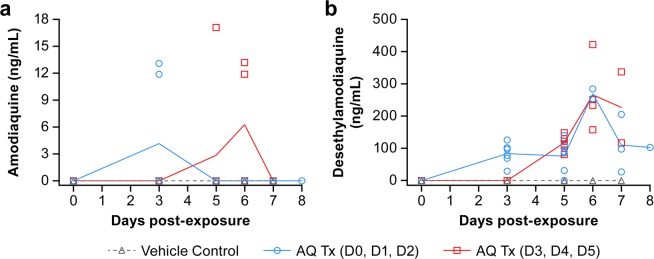
Plasma concentrations of amodiaquine and desethylamodiaquine in EBOV-infected rhesus macaques. Animals were treated orally with vehicle (group 1, black), with 20 mg/kg amodiaquine either on days 0, 1, 2 (group 2, blue) or on days 3, 4, 5 postexposure (group 3, red). Amodiaquine (**a**) and desethylamodiaquine (**b**) concentrations were determined in the plasma at the indicated time points. Results are presented as individual data points with the group means connected by a line. AQ and DEAQ concentrations were adjusted for the dilution factor 5.5.

Gamma-irradiated plasma samples from infected animals collected on days 0, 3, 5, and 7 postexposure and on day of necropsy (days 6, 7 or 8) were analyzed for determination of plasma levels of AQ and its metabolite DEAQ. Data indicate negligible levels of AQ in all but five plasma samples, each from a different animal and day (Fig. 6a**)**. As the samples were diluted by a factor of 5.5 for decontamination protocol, the concentrations were likely below the limit of detection. Plasma levels of the metabolite DEAQ were higher than AQ and detectable in all treated animals from both groups 2 and 3 (Fig. 6b). DEAQ was evident through day 7 postexposure and/or necropsy in 5 of 6 animals in Group 2 and in all animals in Group 3.

**Figure 7:**
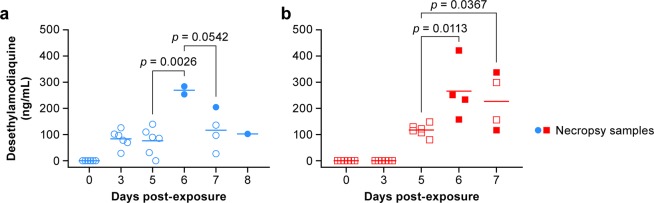
Plasma concentrations of desethylamodiaquine (DEAQ) in EBOV-infected rhesus macaques. DEAQ plasma concentrations of group 2 (**a**; treatment on days 0, 1, 2 postexposure; blue) and group 3 (**b**; treatment on days 3, 4, 5 postexposure; red). The mean on each day is depicted as a horizontal line. Open symbols correspond to scheduled sampling, whereas filled symbols represent necropsy samples. DEAQ concentrations were adjusted for the dilution factor 5.5.

Plasma levels of DEAQ in EBOV-infected NHPs were compared at different time points (Fig. 7). Animals that were treated on days 0, 1 and 2 (Group 2, Fig. 7a), had plasma DEAQ levels ranging from 0 to 205 ng/ml on days 3, 5, 7 and 8 postexposure. The highest DEAQ levels were detected in 2 necropsy samples on day 6 (254 and 284 ng/mL). For animals treated on days 3, 4 and 5 postexposure (Group 3, Fig. 7b), plasma concentrations ranged from 80‒149 ng/mL on day 5. Higher concentrations were detected on day 6 (158‒422 ng/mL) and day 7 postexposure (117‒337 ng/mL).

**Figure 8:**
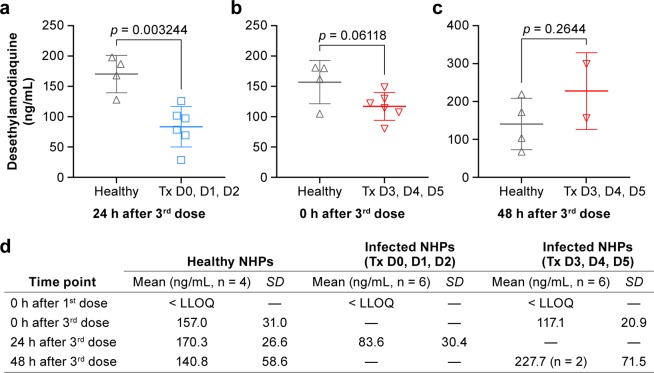
Comparison of desethylamodiaquine concentrations in healthy and EBOV-infected animals. DEAQ plasma concentrations of healthy subjects (black) from the pharmacokinetics study were compared with samples of infected animals in group 2 (treatment on days 0, 1, 2 postexposure; blue) at 24 h after the 3^rd^ dose (**a**), group 3 (treatment on days 3, 4, 5 postexposure; red) at 0 h after the 3^rd^ treatment (**b**), or group 3 at 48 h after the 3^rd^ treatment (**c**). DEAQ concentrations are shown as mean ± SD (**d**). DEAQ concentrations were adjusted for the dilution factor 5.5.

Plasma levels of DEAQ in infected NHPs and healthy NHPs were compared at select time points that were identical in the PK and the efficacy study. Plasma samples from infected Group 2 animals on day 3 correspond to PK-plasma samples taken at 24 h after the 3^rd^ dose. Healthy animals had higher plasma levels of DEAQ (128‒198 ng/mL) than infected animals (29‒126 ng/mL) (Fig. 8a). Similarly, plasma samples on day 5 from infected Group 3 animals were in a lower range (n = 6, 80‒149 ng/mL) than those of healthy animals at the corresponding time point (0 h after 3^rd^ dose, n = 4, 105‒181 ng/mL) (Fig. 8b). On day 7, 48 h after the 3^rd^ dose, DEAQ levels in the plasma of two animals from Group 3 were in a similar range (n = 2, 156‒299 ng/mL) as found in healthy animals (n = 4, 68‒219 ng/mL) (Fig. 8c,d).

## Discussion

Repurposing drugs continues to be of interest to the healthcare professional community for the treatment of emerging and re-emerging hemorrhagic fever viruses such as Ebola, Marburg, and Lassa viruses. Extensive efforts to screen approved and established drugs for antiviral activity led to a panel of drugs with broad-spectrum antiviral activity profiles that are available, affordable, have well-characterized PK/safety profiles and could be used under the Emergency Use Authorization (EUA) mechanism^5,6,13,14^. While a number of FDA-approved compounds have proven to be efficacious against EBOV *in vitro* or in murine models of disease, clinical evaluation or evaluation in more relevant disease models, such as NHPs, has been limited.

AQ is a well-known antimalarial drug with wide usage in the countries that experience outbreaks of EVD. The drug has activity against several human pathogens of other virus families including corona-, flavi-, and bunyaviruses^15–18^. A recent report showed that entry of Lassa-GP pseudotyped virus was blocked by AQ, indicating that the drug may also have value for the treatment of other viral hemorrhagic fevers^19^. AQ is known to have *in vitro* antiviral activity against EBOV^4–8^. Multiple mechanisms for antiviral activity have been proposed. AQ may block EBOV entry, which depends on the acidification of endosomes. As a cationic amphiphilic drug, AQ can accumulate in the late endo/lysosome and, as a weak base, neutralize the acidic environment, thereby inhibiting cathepsin B activation required for fusion of the viral and endosomal membrane^20^. AQ has also been reported to bind and inhibit cathepsin B directly^8^. *In silico* binding studies revealed that AQ may also bind to the viral protein VP35^21^.

We found that AQ inhibits EBOV replication in multiple cell types, including Huh 7 and Vero E6 cells and primary human macrophages, with IC_50_’s ranging from 3.2–27.9 uM depending on experiment and the cell type (Table 1). AQ is rapidly metabolized to the active metabolite, DEAQ, following oral administration. *In vitro* studies confirmed that the active metabolite also has anti-EBOV properties in Huh 7 and Vero E6 cells, and primary human macrophages, with IC_50_’s ranging from 2.8–30.0 µM µM depending on experiment and the cell type. In contrast, AS and its metabolite DHA demonstrated minimal to no *in vitro* antiviral activity against EBOV when tested side-by-side to AQ using our assay parameters. When used in combination, AS and AQ did not have a synergistic effect on EBOV replication *in vitro*. Whereas AS could possibly act with AQ to impact the whole body-system in response to EBOV in a way that cannot be replicated in the *in vitro* assays, this study assumed that AQ was the main contributing factor of the drug combination resulting in anti-EBOV activity.

The goal of the study was to treat animals with AQ using a similar dosing strategy as for human patients, with a target blood concentration range of the parent compound AQ of 29.2 ± 10.9 ng/mL^12^. Indeed, we found that a 20 mg/kg AQ dose using the 3-day treatment regimen resulted in correlating plasma levels of 7.8‒31.9 ng/mL in healthy NHPs. However, this treatment regimen did not result in a survival benefit or decrease of disease symptoms in EBOV-infected animals. These results are disappointing and highlight the importance of utilizing relevant animal models of EVD to evaluate potential antivirals, including detailed characterization of pharmacokinetics and tolerability in the context of EVD. Whereas AQ treatment did not lead to detectable beneficial effects on EVD progression in NHPs, there may be value in investigating novel AQ derivatives that have been improved for anti-EBOV potency and/or improved tolerability^22^.

A possible impact from AQ side effects needs to be considered. Rare instances of acute liver injury usually after prolonged treatment have been reported^23,24^. For this reason, AQ and the combination drug ASAQ are recommended for use as a malaria treatment in endemic areas, but not for prophylaxis against malaria. The onset of hepatic injury is often associated with agranulocytosis. Elevated liver enzymes, ALT and AST, were observed in the healthy NHPs during the pharmacokinetics study.

Given that the EVD patients in 2014 were given a co-formulation of AQ and AS, it would have been of interest to test the combined regimen rather than just AQ to evaluate potential implications of the two drugs on disease progression. Whereas AS has no measurable anti-EBOV effect when tested in combination with AQ *in vitro*, the drug could have an *in vivo* effect that is not captured in cell culture assays. AS and artemether are derivatives of artemisinin and are both metabolized to DHA, the active metabolite for the treatment of malaria. However, AS is water soluble, and reaches peak concentration more rapidly with higher plasma C_max_ than artemether, which could impact *in vivo* activity against EBOV. Another consideration is that AS and AQ may affect each other in terms of PK, drug metabolism, or disposition. For example, AS co-administration with AQ has been reported to reduce exposure to AQ^12^. Questions remain on the possible contribution of AS to the beneficial effect of the ASAQ treatment observed in EVD patients^2^. Therefore, testing the co-formulation would have value, but without testing each drug singly as well, attribution of any observed effect to a specific component will be challenging. Other confounding factors not measured in the Gignoux study could have accounted for the beneficial effect of ASAQ^2^. The potential impact of concurrent malaria and the type of antimalarial used in EVD patients was not addressed in our study. In conclusion, treatment with AQ did not have a beneficial effect on survival or the symptoms of EVD in rhesus macaques under the conditions tested. Whereas AQ on its own failed to demonstrate efficacy, we cannot exclude potential therapeutic value of AQ when used in combination with another antiviral.

## Materials and Methods

### Ethics statement

All animal studies were conducted in facilities accredited by the Association for Assessment and Accreditation of Laboratory Animal Care International (AAALAC) and were approved by either the Institutional Animal Care and Use Committee (IACUC) of SRI International or National Institute of Allergy and Infectious Diseases, Division of Clinical Research, and were in compliance with the Animal Welfare Act regulations, Public Health Service policy, and the *Guide for the Care and Use of Laboratory Animals* recommendations.

### Biosafety

All work with infectious EBOV and potentially infectious materials derived from animals was conducted in a Biosafety Level 4 (BSL 4) laboratory at Integrated Research Facility, National Institute of Allergy and Infectious Diseases (NIAID, Fort Detrick, MD).

### Cells and virus

Vero E6 (ATCC CRL-1586, Manassas, VA), HeLa (ATCC CCL-2), and Huh 7 (human hepatocellular carcinoma) cells were maintained following recommended protocols. Human monocyte-derived macrophages (MDMs) were generated as previously described^25^. Ebola virus/H.sapiens-tc/GIN/2014/Makona-C05 (EBOV/Mak, GenBank accession no. KX000400.1) was propagated in Vero E6 cells (BEI resources, NIAID, NIH: VERO C1008 (E6), African green monkey kidney, Working Bank #NR-596) as previously described^26^. Virus stock and challenge inoculum titers were determined by plaque assay on Vero E6 cells as previously described^26^.

### Drugs and treatment preparation

*In vitro studies*. The antimalarial artesunate-amodiaquine (ASAQ Winthrop) (Sanofi-Aventis, Gentilly Cedex, France) was solubilized by crushing the tablets and resuspending in dimethyl sulfoxide to prepare a solution with the AQ component at a concentration of 100 mM. The compounds, amodiaquine dihydrochloride dihydrate (#A2799), artesunate (#A3731), and dihydroartemisinin (#D7439) were purchased from Sigma-Aldrich (Saint Louis, MO). N-desethylamodiaquine hydrochloride (#Sc-212178) was obtained from Santa Cruz Biotechnology (Dallas, TX). Drugs were prepared as 100 mM stocks in dimethyl sulfoxide. *Animal studies*. Source, formulation, and preparation of amodiaquine hydrochloride was the same for the PK and the efficacy studies. Amodiaquine hydrochloride was obtained from US Pharmacopeia (Rockville, MD; Cat. 1031004; Lot J01144) and a 4 mg/mL AQ (free base) solution was prepared in Sterile Water for Injection (USP).

### Cell-based testing of EBOV antiviral agents

The cell-based EBOV drug screen and cytotoxicity assays were performed as previously described^25^. Briefly, Vero E6 and Huh 7 cells were seeded at 3 × 10^4^ cells/well, and MDMs at 1 × 10^5^ cells/well in 96-well plates. After 24 h, cells were treated with compounds at 2-fold dilutions starting from 40 µM. The starting concentration of the ASAQ tablet suspension corresponded to 40 µM of the AQ base component. Cells were infected with EBOV/Mak 1 h after the addition of the drugs in biosafety level 4 (BSL4) containment at multiplicity of infection (MOI) of 0.2–0.5. After 48 h, plates were fixed with 10% neutral buffered formalin (Richard-Allan Scientific), and EBOV/Mak was detected with a mouse antibody specific for EBOV VP40 protein (#B-MD04-BD07-AE11, USAMRIID)^27^ followed by staining with Alexa Fluor® 594 goat anti-mouse IgG (heavy + light chain) antibody (Life Technologies, Grand Island, NY) or with anti-mouse IgG-peroxidase labeled antibody (KPL#074–1802). Fluorescence or luminescence was quantified on a plate reader (Infinite® M1000 Pro, Tecan US, Morrisville, NC). The signal of treated infected wells was normalized to uninfected control wells and measured (in percent) relative to untreated infected wells.

Non-linear regression analysis was performed, and the 50% inhibitory concentrations (IC_50_s) were calculated from fitted curves (log [agonist] versus response [variable slope] with constraint to remain above 0%) (GraphPad Software, La Jolla, CA). The EBOV drug screen assay was performed with three replicates for each drug concentration, and the assay was repeated at least twice for confirmation.

To evaluate cytotoxicity, cells were treated with compounds as described above in absence of virus. At 48 h after drug addition, cell viability was quantified using the CellTiter Glo luminescent cell viability assay kit (Promega, Madison, WI).

### Pharmacokinetic analysis of amodiaquine in nonhuman primates

Rhesus macaques (*Macaca mulatta*) were obtained from Covance Research Products (Princeton, NJ). Two groups (n = 4, 2 females and 2 males per group) were dosed with 20 or 40 mg/kg amodiaquine hydrochloride (US Pharmacopeia, Rockville, MD; Cat. 1031004; Lot J01144) orally via nasogastric intubation once daily for 3 days. NHPs were housed in stainless steel primary enclosures singly or in pairs if compatible. NHPs were provided Teklad Certified Global 20% Protein Primate Diet (#2050c) and purified water ad libitum. Clinical observations were conducted for 5 days including the day of dosing. Blood (maximal 500 μL) was collected from cephalic vessels on day 1 and day 3. On day 1, blood was collected pre-dose, 0.5, 1, 2, 4, 6, 8, 12, and 24 h post-dose (immediately prior to dosing on day 2). On day 3, blood was collected 24 h after dose 2 (or 48 h after dose 1), immediately prior to dosing on day 3, and 0.5, 1, 2, 4, 6, 8, 12, 24 and 48 h after dose 3.

Drug concentrations of AQ and its metabolite, DEAQ, were determined in collected plasma samples using liquid chromatography with tandem mass spectrometry (LC-MS/MS). Plasma samples (volume 50 μL) were prepared by adding 1 mL of methyl t-butyl ether. Each tube was vortexed for approximately 15 minutes (min) at maximal speed and centrifuged for 10 min at 18,000 *g* to facilitate separation of the liquid phases. Upper layers (850 μL) were transferred to new tubes, and the solvent was removed under vacuum in a centrifugal evaporator. The dried residues were reconstituted with 100 μL of internal standard solution (400 ng/mL risperidone and 400 ng/mL chloroquine in 75:25 [v:v] acetonitrile:water). The tubes were vortexed 3 min and clarified by centrifugation (18,000 *g*) for 3 min. The clarified extracts were transferred to high performance liquid chromatography vials containing glass inserts for subsequent LC-MS/MS analysis. LC-MS/MS was performed using a LC-20AD pump system (Shimadzu, Columbia, MD) and a 4000 QTrap mass spectrometer (Sciex, Concord, Ontario, Canada) in multiple reaction monitoring mode and a Polaris C18-A column (50 × 2.1 mm, 5 μm; Agilent, CA), using gradient elution with 0.1% formic acid in water and 0.1% formic acid in acetonitrile as the mobile phase.

The lower limit of quantitation for AQ and DEAQ of the method was 5 ng/mL. The following parameters and constants were determined: maximal plasma concentration (Cmax), time to maximum plasma concentration (Tmax), area under the plasma concentration-time curve to the last time point and extrapolated to infinity (AUC_last_ and AUC_inf_), terminal elimination half-life (t1/2), apparent volume of distribution (V/F), and total clearance (Cl/F) after oral administration. PK parameters were analyzed using Phoenix^®^ WinNonlin^®^ software (v 6.3; Certara, Princeton, NJ) to perform noncompartmental data analysis for extravascular administration. For clinical pathology (serum chemistry, hematology), blood (1 mL) was collected prior to dosing on day 1 and approximately 24 h after the final day 3 dose.

### Treatment and challenge of nonhuman primates

Rhesus macaques of Chinese origin (n = 15, adult, <10 years of age) were obtained from Charles River Laboratories (Frederick, MD). Animals were singly housed in stainless steel primary enclosures and provided chow and purified (reverse osmosis) water *ad libitum*. The vehicle control group (group 1; n = 3) was treated with sterile water. Two treatment groups were treated with AQ orally once daily as a consecutive 3-day course of 20 mg/kg free base (26 mg/kg amodiaquine hydrochloride (US Pharmacopeia, Rockville, MD; Cat. 1031004; Lot J01144). Treatment began on the day of EBOV exposure (group 2; n = 6, 3 females and 3 males), or on day 3 postexposure (group 3; n = 6, 3 females and 3 males). All groups were challenged IM with a target dose of 1000 PFU) of EBOV/Mak (actual dose = 1080 PFU). The plaque assay back-titration of the challenge material was initiated on the day of preparation (study day 0) on Vero E6 cells, and plaques were fixed with 10% formalin and stained with crystal violet 8 days later. Animals were observed and weighed daily. The vehicle control group (group 1, n = 3) received an equivalent volume of sterile water (Gibco, Cat. A1287302, Lot 1835987).

Weight was recorded on day -12 and day -7, then daily starting one day before challenge until primates succumbed to disease. Responsiveness of the animals was monitored following 5-point range, in which a score of 4 met primary endpoint criteria and 3 initiated secondary criteria evaluation (Table 2). When NHPs scored a 3 for primary euthanasia criteria and their temperature was above 34 °C, secondary euthanasia criteria were reviewed. An NHP had to meet at least two secondary criteria, blood urea nitrogen ≥ 68 mg/dL, calcium ≤ 6.8 mg/dL, GGT ≥ 391 U/dL, and/or creatinine ≥ 2.8 mg/dL, for required euthanasia. Two rhesus macaques were euthanized based on their secondary criteria. Six of the fifteen rhesus macaques were euthanized based on veterinary discretion, not based on their clinical score or secondary criteria.

### Hematology and serum chemistry

Complete blood count with leukocyte differential was performed from peripherally collected blood samples using Vacuette K_3_ ethylenediaminetetraacetic acid (EDTA) tubes and a Sysmex XT-2000iV hematology instrument using a preprogrammed monkey species profile (Sysmex America, NY). Plasma and serum were prepared by separation for 10 min at ambient temperature with centrifuge set to 1800 RCF. Serum chemistries were performed using the Piccolo Xpress Analyzer and General Chemistry 13 discs (Abaxis, Abbott Point of Care, NJ) from Vacuette Z Serum Clot Activator tubes (Greiner Bio-One, Monroe, NC).

### Pathology and histology

All animals were humanely euthanatized in accordance with defined experimental endpoints, and gross necropsy was performed. Tissue samples were inactivated by fixing for 72 h in 10% neutral buffered formalin. Following fixation and removal from the BSL-4 laboratory in accordance with standard operating procedures, tissue samples were routinely processed in a Tissue-Tek VIP-6 automated vacuum infiltration processor (Sakura Finetek USA, Torrance, California, USA) followed by paraffin embedding with a Tissue-Tek model TEC unit (Sakura Finetek USA, Torrance, CA, USA). Using a standard semiautomated rotary microtome and lighted water flotation bath (Leica Biosystems, Wetzlar, Germany), tissue sections were cut at a thickness of 4 µm and mounted on positively charged uncoated glass slides (ThermoFisher, Waltham, MA, USA), air-dried at room temperature, stained with hematoxylin and eosin (H&E), and coverslipped for microscopic evaluation by the pathologist.

### Viral load evaluation

#### Viral load determination using quantitative real time RT-PCR

Plasma was inactivated in TRIzol LS (ThermoFisher Scientific, Waltham, MA), and total RNA was isolated using the QIAamp Viral RNA Mini Kit (QIAGEN, Germantown, MD) in accordance with manufacturer’s instructions. Briefly, 70 μL of TRIzol LS-inactivated sample was added to 280 μL of QIAGEN Buffer AVL containing carrier RNA. Sample was eluted in 70 μL of Buffer AVE, aliquoted, and frozen until assayed. Plasma viral RNA was quantified using BEI Resources Critical Reagents Program EZ1 RT-qPCR (TaqMan) assay kit on an ABI 7500 FastDx (Applied Biosystems, ThermoFisher Scientific) in accordance with manufacturer’s instructions using a synthetic RNA standard curve and reported as viral RNA copies (log_10_) per mL of sample.

#### Virus titration by plaque assay

Plasma samples were diluted in Dulbecco’s modified eagle medium with GlutaMAX (Gibco) and 10% HI-FBS (Gibco) and added to confluent monolayers of Vero E6 cells in triplicate. Samples were adsorbed to the cell monolayers for 1 h at 37 °C and 5% CO_2_ with gentle rocking approximately every 15 min to prevent monolayer drying. The monolayers were then overlaid with a 1:1 mix of 2.5% Avicel RC 591 biopolymer (FMC BioPolymer) mixed with 2X Modified Eagle Medium (Temin’s modification, Gibco) supplemented with 2X Antibiotic-Antimycotic (Gibco), 2X GlutaMAX and 10% HI-FBS. Cells were incubated at 37 °C and 5% CO_2_ for 8 days, stained and fixed with 0.2% aqueous Gentian Violet (Ricca Chemicals) in 10% neutral buffered formalin for 30 min, rinsed, and plaques were enumerated.

### Pharmacokinetic analysis of amodiaquine in plasma samples of Ebola virus-infected animals

Plasma samples from rhesus macaques that had received a dose of AQ at Integrated Research Facility/NIAID (Fort Detrick, MD) were analyzed for concentrations of AQ and the metabolite DEAQ. Prior to shipment to SRI, the samples were diluted 1:5.5 and irradiated at 5 Mrad using a cobalt-60 source to destroy any pathogens that may have been present in the plasma samples. A pilot study with spiked quality control standards confirmed that irradiation does not appreciably alter the level of AQ in the plasma (data not shown). A liquid:liquid extraction method with methyl t-butyl ether was used to separate AQ and DEAQ from rhesus macaque plasma. Samples were analyzed by LC-MS/MS with a lower limit of quantitation (LLOQ) of 10 ng/mL (2.00 ng/mL × 5.5 dilution factor) in plasma for both AQ and DEAQ. AQ and DEAQ concentrations were adjusted for the dilution factor of 5.5 for analysis in Figs. 7 and 8.

## Supplementary information

Supplementary Information

## Data Availability

The virus working stock EBOV/Makona used for exposure (GenBank accession no. KX000400.1, BioSample: SAMN04490241).
