# Supplementary material for: In Vivo Activity of Amodiaquine against Ebola Virus Infection
Source: Sci Rep. 2019 Dec 27;9:20199. doi: 10.1038/s41598-019-56481-0 (PMC6934550; doi:10.1038/s41598-019-56481-0)

In Vivo Activity of Amodiaquine against Ebola Virus Infection

L. Evans DeWald, J. C. Johnson, D. M. Gerhardt, L. M. Torzewski, E. Postnikova, A. N. Honko, K. Janosko, L. Huzella, W. E. Dowling, A. E. Eakin, B. L. Osborn, J. Gahagen, L. Tang, C. E. Green, J. C. Mirsalis, M. R. Holbrook, P. B. Jahrling, J. Dyall & L. E. Hensley

Supplementary Information

In Vivo Activity of Amodiaquine against Ebola Virus Infection

L. Evans DeWald, J. C. Johnson, D. M. Gerhardt, L. M. Torzewski, E. Postnikova, A. N. Honko, K. Janosko, L. Huzella, W. E. Dowling, A. E. Eakin, B. L. Osborn, J. Gahagen, L. Tang, C. E. Green, J. C. Mirsalis, M. R. Holbrook, P. B. Jahrling, J. Dyall & L. E. Hensley

Supplementary Table 1: Pharmacokinetic parameters for amodiaquine in healthy rhesus macaques after oral dose administration.

Animal No.	Day	Sex	Dose (mg/kg)	T_{max} (h)	C_{max} (ng/ml)	$t_{1/2}$ (h)	AUC_{last} (hr·ng/ml)	AUC_{inf} (hr·ng/ml)
1	1	M	20	8	7.84	NC[a]	48	NC[b]
2	1	M	20	6	9.19	NC	44	NC
3	1	F	20	2	31.9	35.9	128	837
4	1	F	20	4	19.3	NC	76	NC
			Mean	*5*	*17.1*	*35.9*	*74*	*837*
			SD	*3*	*11.1*	*NA*	*39*	*NA*
5	1	M	40	8	12.6	NC	189	NC
6	1	M	40	6	19.0	4.3	138	184
7	1	F	40	4	29.0	3.5	172	200
8	1	F	40	2	154	3.0	532	590
			Mean	*5*	*53.7*	*3.0*	*258*	*324*
			SD	*3*	*67.2*	*0.6*	*184*	*230*
1	3	M	20	4	15.1	NC	96	NC
2	3	M	20	6	11.7	NC	107	NC
3	3	F	20	0.5	47.4	3.1	195	236
4	3	F	20	1	14.5	4.8	10 to	136
			Mean	*3*	*22.2*	*4.8*	*133*	*186*
			SD	*3*	*16.9*	*NA*	*54*	*NA*
5	3	M	40	6	19.6	28.4	349	849
6	3	M	40	6	32.2	12.9	409	579
7	3	F	40	4	35.1	3.6	243	281
8	3	F	40	2	28.2	10.4	351	447
			Mean	*5*	*28.8*	*10.4*	*338*	*539*
			SD	*2*	*6.7*	*10.5*	*69*	*240*

[a]NA, not applicable; [b]NC, not calculated because elimination phase did not exhibit sufficient linearity

In Vivo Activity of Amodiaquine against Ebola Virus Infection

L. Evans DeWald, J. C. Johnson, D. M. Gerhardt, L. M. Torzewski, E. Postnikova, A. N. Honko, K. Janosko, L. Huzella, W. E. Dowling, A. E. Eakin, B. L. Osborn, J. Gahagen, L. Tang, C. E. Green, J. C. Mirsalis, M. R. Holbrook, P. B. Jahrling, J. Dyall & L. E. Hensley

Supplementary Table 2: Pharmacokinetic parameters for desethylamodiaquine in healthy rhesus macaques after oral dose administration

Animal No.	Day	Sex	Dose (mg/kg)[a]	T_{max} (h)	C_{max} (ng/ml)	$t_{1/2}$ (h)	AUC_{last} (h·ng/ml)	AUC_{inf} (h·ng/ml)
1	1	M	20	8	203	15	3,280	5,275
2	1	M	20	8	290	12	3,999	5,990
3	1	F	20	8	369	NC[b]	5,996	NC
4	1	F	20	4	287	16	3,579	5,734
			Mean	*7*	*287*	*15.6*	*4,214*	*5,666*
			SD	*2*	*68*	*1.7*	*1,224*	*362*
5	1	M	40	8	435	NC	7,241	NC
6	1	M	40	6	441	14	5,958	9,647
7	1	F	40	4	515	10	7,551	9,948
8	1	F	40	2	1,080	15	10,146	14,235
			Mean	*5*	*618*	*15.0*	*7,724*	*11,277*
			SD	*3*	*310*	*2.5*	*1,756*	*2,566*
1	3	M	20	1	453	22	10,805	14,126
2	3	M	20	8	383	51	10,681	23,278
3	3	F	20	4	660	13	14,320	18,388
4	3	F	20	1	527	18	8,238	10,032
			Mean	*3*	*506*	*18.3*	*11,011*	*16,456*
			SD	*3*	*118*	*16.9*	*2,502*	*5,685*
5	3	M	40	8	618	36	20,369	33,147
6	3		40	6	541	40	16,500	29,023
7	3	F	40	4	846	38	17,591	28,023
8	3		40	2	538	43	17,427	31,817
			Mean	*5*	*636*	*42.8*	*17,972*	*30,502*
			SD	*3*	*145*	*3.0*	*1,669*	*2,385*

[a]Dose of amodiaquine; [b]NC, not calculated because elimination phase did not exhibit sufficient linearity

Supplementary Figure 1

In Vivo Activity of Amodiaquine against Ebola Virus Infection

L. Evans DeWald, J. C. Johnson, D. M. Gerhardt, L. M. Torzewski, E. Postnikova, A. N. Honko, K. Janosko, L. Huzella, W. E. Dowling, A. E. Eakin, B. L. Osborn, J. Gahagen, L. Tang, C. E. Green, J. C. Mirsalis, M. R. Holbrook, P. B. Jahrling, J. Dyall & L. E. Hensley

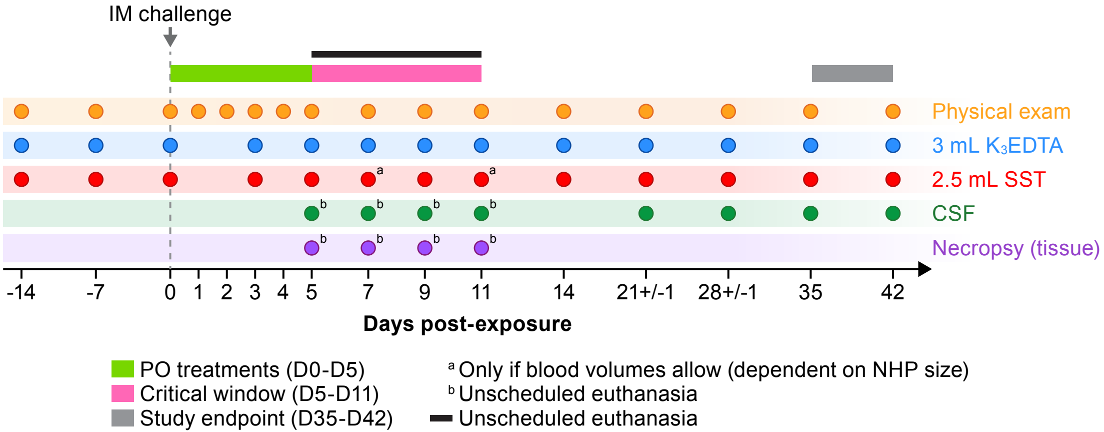

Supplement: Supplementary file 1 — Supplementary Information [file 41598_2019_56481_MOESM1_ESM.pdf]
